# Perceiving object size in pictures involves high-level processing

**DOI:** 10.1098/rspb.2024.2967

**Published:** 2025-05-14

**Authors:** Ecem Altan, H. Boyaci, Steven C. Dakin, D. Samuel Schwarzkopf

**Affiliations:** ^1^ School of Optometry & Vision Science, The University of Auckland, Auckland, New Zealand; ^2^ Departments of Psychology and Neuroscience, Bilkent University, Ankara, Türkiye; ^3^ A.S. Brain Research Center & National Magnetic Resonance Research Center (UMRAM), Bilkent University, Ankara, Turkey; ^4^ Department of Psychology, Justus Liebig University Giessen, Giessen, Hessen, Germany; ^5^ Institute of Ophthalmology, University College London, London, England, UK; ^6^ Experimental Psychology, University College London, London, England UK

**Keywords:** perceived size, top-down processing, inverted Ponzo illusion, population receptive field, fMRI

## Abstract

Spatial context is critical for telling how big a visual object is, although it may also cause the perceived size to diverge dramatically from the true dimensions. Interestingly, responses in the primary visual cortex (V1) mirror such illusory perception; however, the stage of processing that leads to such neural correlates remains unknown. Here, we tested the involvement of higher level processing in a Ponzo-like illusion, by quantifying the effect of manipulating depth cues and inversion of the whole scene. We report a stronger illusion for realistic compared with simpler backgrounds, and for upright compared with inverted scenes (except for scenes where the target objects appeared on the ceiling or in the sky). Next, using functional MRI, we tested the effect of inversion on V1 responses. Inverted scenes elicited a smaller extent of activation in V1 compared with upright scenes, consistent with their perceived sizes. Taken together, since the inversion should disrupt the high-level processing while keeping the low-level features intact, our findings demonstrate that Ponzo-like illusions involve high-level processes that integrate contextual depth cues and visual experience, thereby modulating the object’s neural representation in V1.

## 1. Introduction

**Figure 1.**
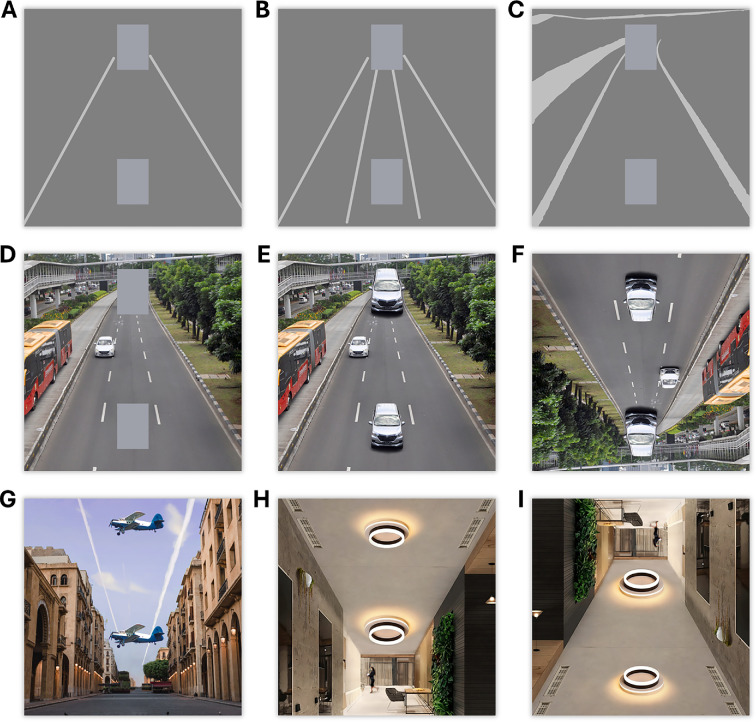
Illusion stimuli used in the psychophysical experiment. The target objects (rectangles in A–D, cars in E–F, planes in G and lamps in H, I) have the same size but appear different to most observers. Inverted versions of road and ceiling conditions are shown in F and I. See electronic supplementary material for side-by-side visual comparisons of all conditions with their inverted version.

The perceived size of objects strongly depends on contextual information rather than their retinal size alone. This is necessarily the case because determining object size from retinal size is an under-constrained problem, since distance and size are confounded. Furthermore, limited cues in flat images compromise our ability to estimate veridical size. The Ponzo illusion and its variants, including corridor, hallway or railroad illusions and their realistic versions (figure 1), demonstrate this intricate interplay between object size and surroundings. Two identical objects (e.g. cars in figure 1E) in each panel appear to have different sizes although they are the same angular size. How the contextual elements in these illusions influence size perception has been studied extensively over the past century; nevertheless, the mechanism leading to this misestimation of image size remains poorly understood.

While these illusions are often associated with implied depth in the background [1–3], some low-level/feature-driven mechanisms have also been suggested. One such bottom-up explanation is that the boundary elements are being *assimilated* towards the surrounding elements that fall within the attentive field, expanding the area of the target [4,5]. This hypothesis predicts size overestimation of the upper stimulus (e.g. figure 1A) but no change in the lower stimulus due to a lack of nearby elements in the attentive field. Another proposed explanation is that the perceived stimulus size is influenced by the relative sizes of neighbouring elements [6,7]. Based on this reasoning, the upper stimulus looks larger relative to the small space around it, and the lower one looks smaller relative to the wide empty area around it. These accounts, and some others that are solely based on stimulus features (e.g. [8,9]; see [10] for a review), were originally proposed to explain the classical Ponzo illusion, but can be theoretically applied to some richer variants of the Ponzo illusion as well.

Explanations based on perceived relationships of objects in depth suggest that the angled inducers imply depth in the image and lead to misapplied size constancy [2]. This theory predicts that the perceptual effect should increase as the availability of depth-related cues increases. Consistent with this view, the illusion increases when the scene contains more texture elements, perspective and interposition cues [1,11]. This account also posits that the effect relies on the observer’s prior experience of, and internal expectations about, the size and distance relationship. Therefore, the strength of the effect should vary based on the similarity of the image to the observer’s visual experience. Developmental [12] and cross-cultural [1] studies have supported these predictions.

Here, we aimed to test whether the low-level stimulus interactions can explain the illusion and the corresponding cortical activity in the primary visual cortex (V1), or whether feedback from high-level processing plays a significant role. To test this, we used another critical manipulation in the investigation of these size illusions, which is the inversion of the illusion images. Inversion ensures that low-level stimulus-related effects, such as geometrical properties, contour interaction and contrast, remain constant, while the high-level interpretative aspects, such as implied depth and scene familiarity, are disrupted. Low-level features here refer to the visual properties processed at the initial stages through bottom-up mechanisms, where elements like lines, edges and colours are analysed without top-down influences from prior knowledge or expectations about the objects being viewed. If the magnitude of the effect remains unchanged after inversion, it suggests that the perceptual effect arises solely through an interaction between the low-level stimulus features. Conversely, if the illusion diminishes or changes, this supports the involvement of high-level processing. To date, only a few studies have systematically investigated the effects of image rotation on the Ponzo-like illusions [13–15]. Richards & Miller [15] reported that the perceptual effect induced by a background grid forming a corridor decreased when the background was inverted. Similarly, Poom [14] tested the Ponzo illusion in eight orientations and demonstrated that the illusion was stronger when presented upright (5.6% illusion magnitude) than 180${,}^{\circ }$ inverted (3.2%), and that the correlation between different orientations, on average, decreased with increasing orientation difference.

Inversion could also provide crucial insights into the mechanism involved in the cortical representation of object size within such illusions. Illusory size increase in a Ponzo-like illusion is accompanied by an increased area of response in V1 [16–18]. This is thought to result from a feedback process that modulates V1 activity because (i) the receptive fields of V1 are too small to process the entire scene and (ii) the extent of activation has been shown to be modulated by visual attention [19]. Supporting this hypothesis, previous studies showed that size processing involves high-level cortical regions [20,21]. However, the perceptual illusion might result from both the depth-related and low-level mechanisms discussed above. As such, the V1 response to such illusions might only reflect the low-level component. In the same vein, we recently showed that V1 activity reflects the perceived separation of stimuli in the Mueller-Lyer illusion; however, in this case, that is presumably a simple consequence of spatial filtering by V1 neurons [22]. Since inversion disrupts high-level (top-down) rather than low-level (bottom-up) processing of the Ponzo illusion, if V1 activity is unchanged between upright and inverted illusions, we can therefore postulate that V1 activity only reflects the low-level, feed-forward components of this illusion. Conversely, if there is a change in V1 activity after inversion, this would provide direct evidence that feedback from higher regions modulates the cortical representation of the object in V1.

Here, we tested the involvement of high-level mechanisms in the Ponzo illusion using both behavioural experiments and functional brain imaging. In psychophysical experiments, we investigated the role of varying levels of depth cues and the role of different photorealistic environments in the illusion magnitude and, importantly, whether the inversion of the scene modulates these effects. Furthermore, we used functional magnetic resonance imaging (fMRI) to test the effect of depth-inducing background and the effect of scene inversion on the V1 activity. We reconstructed the cortical activation for the car stimuli on the visual field by utilizing population receptive field (pRF) mapping.

## 2. Behavioural experiment

Behavioural experiments were conducted to test whether the image inversion modulates the magnitude of the perceptual effect across conditions containing (i) various levels of depth cues, and (ii) different photorealistic visual environments. We expected to observe weaker perceptual effects for images containing less depth information and an overall weaker perceptual effect for the inverted images, as they are less likely to trigger size constancy than the familiar upright scenes.

### (a) Methods

#### (i) Participants

Sixteen participants (5 males, 11 females; age range: 18−41; M=28) with normal or corrected-to-normal vision volunteered for the experiment. The sample size was based on previous behavioural studies on similar size illusions (e.g. [3]) and initial pilot experiments.

#### (ii) Stimuli and apparatus

Seven sets of stimuli were used in the experiment, including both photographic and abstract visuals. The *road* condition involved cars on a road picture (figure 1E), which formed a base for the abstract versions with various levels of depth cues: rectangles on classical Ponzo lines (*Ponzo*, figure 1A), rectangles on four lane lines (*lanes*, figure 1B), rectangles on a simplified road scene (*figurative*, figure 1C), and rectangles on a road photo (*hybrid*, figure 1D). Additionally, two more photographic images were used, consisting of planes in the sky (*sky*, figure 1G) and lamps on a ceiling (*ceiling*, figure 1H). Each stimulus set was presented in upright and inverted (upside-down) configurations. Inverted versions were obtained by rotating the background and the target stimuli by 180${,}^{\circ }$.

The photograph used in the road condition was a stock image [23], rotated and cropped to ensure that the road portion of the background image was centred and the geometric centre of the target stimuli positions remained the same in both rotation conditions. The image was modified further to obtain the simpler versions: the figurative background was obtained by selecting a few main shapes that provide depth information, and the lanes and Ponzo conditions were obtained by simple lines corresponding to the main road lines. We aimed to progressively decrease the high-level properties of the road image in steps. The backgrounds in these conditions were set to mid-grey, the lines and shapes to light-grey and the target stimuli to grey with a blue tint.

The ceiling image was acquired from a retail store website [24], and the plane and the sky images were from stock image websites [25,26]. The ceiling and sky photos were modified to make the angular perspective similar to that of the inverted version of the road image. This similarity was achieved by spreading out structural elements (e.g. walls and buildings) horizontally without altering the true physical perspective cues. The resulting empty areas along the vertical centre of the images were filled in using the generative artificial intelligence tool, together with other standard tools in Adobe Photoshop 25.1 (on Windows). Also, a few elements were added to the ceiling image to prevent the ceiling from being perceived as a floor in its inverted version. These were mostly things that could imply gravitational direction, such as a hanging plant, a droplight and a human walking, as well as ventilation grills, which are commonly on the ceiling rather than on the floor.

The background images were presented at the centre of a mid-grey screen, via MATLAB (v. 2020b, Mathworks), Psychophysics toolbox [27]. The two target stimuli always appeared at the vertical centre of the background image. The participants were seated 65 cm in front of a 27 inch ViewSonic XG270 LCD monitor (VS17961; 240 Hz refresh rate; 1920×1080 pixels screen resolution) in a dark room. A chin rest was used to stabilize the participants’ heads.

#### (iii) Procedure

**Figure 2.**
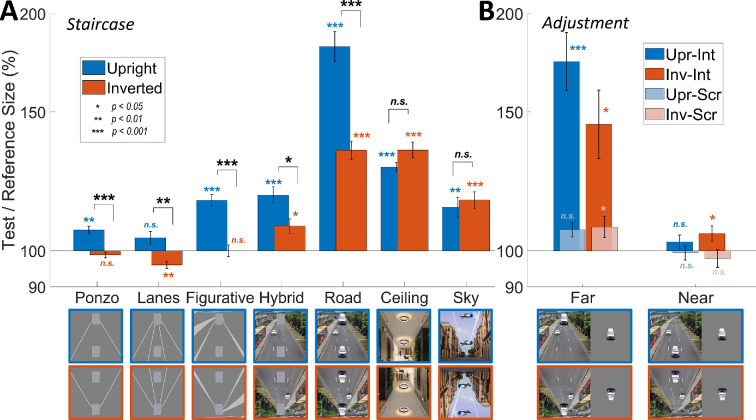
Behavioural results. The magnitude of the illusion as measured in (A) the main psychophysics experiment, and (B) adjustment experiment in the scanner. The *y*-axis represents the group average of the per cent ratio between the test and reference stimulus size (height). Error bars represent standard error across participants. Statistical significance after FDR correction is indicated by coloured (illusory effects) and black (inversion effects) asterisks. Upr: upright, Inv: inverted, Int: intact, Scr: scrambled.

An experimental session consisted of seven separate blocks designated for illusion conditions. These seven illusion blocks were presented in a fixed order, from the most simple background to those richer in depth-related information, as they appear in figure 2A, from left to right. This ordering ensured that the more depth-inducing images did not prime the low-level conditions.

Blocks started with an instruction display that presented the upcoming illusion condition and its corresponding target stimuli (as shown in figure 1). The instruction display also included two red frames around the two target stimuli, which served as a cue to signal participants what to look for in the experimental trials (eliminating possible confusion with other objects in the road condition). Participants were first asked to read the instructions thoroughly and then initiate the experiment with a key press when they were ready. The instruction was to compare the sizes of two target stimuli and report the larger one using the keyboard’s up or down arrow keys.

Illusion blocks consisted of four sub-blocks for the combination of two rotation conditions and two positions (far and near). For each sub-block, one of the two target stimuli, at either far or near position, was fixed throughout the sub-block (reference stimulus), while the one at the other position (test stimulus) changed in size between trials depending on participant responses. The reference stimulus size was fixed, and the size of the test stimuli was subject to a one-up, one-down adaptive staircase procedure. Each measurement had two staircases, starting from either substantially larger or substantially smaller than the reference stimulus. The initial step size was 16% and was halved after each of the first three response reversals. Staircases were presented in a random order.

The stimuli in the experimental trials were run for 700 ms before participants were required to respond. After a valid response was registered, the following trial appeared after a 300 ms inter-trial interval.

All participants were tested over 1680 trials in total ((7 illusions) × (2 rotations) × (2 positions: near and far) × (2 staircases) × (30 trials)).

#### (iv) Data analysis

We reversed the trial responses for the sub-blocks where the reference stimulus appeared at the near position so that the responses reflect whether the far stimulus was perceived as larger than the near (instead of whether the test stimulus was perceived as larger than the reference stimulus). We then pooled all data belonging to the same condition (two staircases from the two test positions). It is worth noting that we combined data from near and far reference car positions, as both measurements reflect the relative size at the two positions and are thus interdependent.

The pooled data were fit with a cumulative log-normal distribution using Psignifit 4 MATLAB Toolbox [28]. The lapse and guess rate parameters were fixed at 0.01. Half-proportion of the fitted functions were acquired as the point of subjective equality (PSE) values. The PSE values here correspond to the ratio between the sizes (height) of the test stimuli and the reference stimuli on a logarithmic scale.

### (b) Results

Figure 2A represents the magnitude of each illusion condition for both rotation conditions, averaged across participants. A value of 100% on the y-axis represents no difference between the perceived sizes of the two target stimuli. Values higher than 100% mean an overestimation of the perceived size of the far stimulus compared to the near.

We found the most substantial perceptual effect for the upright road condition where the far stimulus was perceived as 82% larger than the near stimulus on average. Also, as expected, the Ponzo and the lanes conditions showed weak perceptual effects (6% and 4%, respectively) relative to other conditions containing more depth cues (16% for figurative and 18% for hybrid). Importantly, all conditions, except ceiling and sky, showed a significant decrease (black asterisks) in the illusion magnitude for the inverted conditions compared with their upright counterparts, even in the simplest versions, including the Ponzo illusion. Interestingly, ceiling and sky conditions subtly demonstrated the opposite effect.

The perceptual effect (figure 2A) for the upright version of all seven illusions, except lanes (p=0.2), was significantly different from zero at p<0.01 (blue asterisks). For the inverted conditions, all seven illusion conditions, except for the figurative and Ponzo backgrounds (respectively, p=3.2 and p=0.5), resulted in significant perceptual effects at p<0.05 (orange asterisks; all p-values were FDR-corrected). Surprisingly, inverted lanes condition showed an opposite illusory effect.

There was a main effect of the illusion condition (repeated measures ANOVA: F_2.6, 39.5 _= 112.8, p<0.001, Greenhouse–Geisser corrected) and of rotation (F_1, 15 _= 68.4, p<0.001) on the illusion magnitude, as measured by PSE values. There was also a significant interaction between the two dependent variables (F_3, 45.5 _= 17, p<0.001, Greenhouse–Geisser corrected).

We also tested the inversion effects separately for illusion conditions. Except for the sky and ceiling backgrounds (respectively p=1.6 and p=0.26), there were significant differences between upright and inverted versions for all illusion conditions (paired-samples t-tests, p<0.01, all ps FDR-corrected).

Finally, we found strong correlations between the perceptual effects observed in the opposite rotation conditions of the road background and other realistic images, specifically, inverted road and upright sky (r(15)=0.65, p<0.01), inverted road and upright ceiling (r(15)=0.7, p<0.01) and inverted ceiling and upright road (r(15)=0.64, p<0.01). A full correlation matrix is available at the OSF link https://osf.io/cfqt6/ [29].

## 3. fMRI experiment

Next, we investigated the role of high-level processing on previously reported (e.g. [17]) neural correlates of illusory size perception with Ponzo-like illusions. Specifically, by comparing the neural responses to upright and inverted illusion conditions, we aimed to test whether the V1 activity is solely driven by low-level components of the Ponzo-like illusion, or influenced by high-level feedback.

### (a) Methods

#### (i) Participants

Ten participants (three males, seven females; age range: 23−30; M=26.7) with normal or corrected-to-normal vision volunteered for the fMRI experiment. The sample size was determined based on previous fMRI studies using similar methods [22,30] and similar size illusions [17].

#### (ii) Stimuli and procedure

Stimuli were presented on a 32-inch MRI-compatible LCD monitor (TELEMED Solutions Istanbul, 1360 × 768 pixels resolution, 60 Hz refresh rate) placed at the back of the scanner bore. Participants observed the stimuli through a mirror mounted on the head coil. The monitor’s height subtended 10.6${,}^{\circ }$ of the visual angle from a viewing distance of 213 cm.

Stimuli were presented via MATLAB (v. 2021b, Mathworks) and Psychtoolbox [27]. We used only the road condition (figure 1E) in the fMRI experiment, as it showed the maximum perceptual effect in our psychophysical experiments. The background image was either the road image (*intact*) or a phase-scrambled version of the road as a control condition (*scrambled*). Geometric centres of the car stimuli were placed either at 1/4 or 3/4 height of the image to match the cars’ positions between the upright and inverted conditions. The participants responded using an MRI-compatible response box with four buttons.

An MRI session consisted of the following components in the specified order: a method of adjustment experiment, a structural scan, six experimental runs and three pRF runs.

#### Adjustment experiment

Before scanning, participants completed a brief adjustment experiment in the scanner. This experiment was carried out (i) to ensure that the perceptual effect and the inversion effect were preserved in the scanner environment and (ii) to test whether the control background produced any perceptual effect. The adjustment experiment started with an instruction display that introduced the stimulus and the task to the participants. Participants initiated experimental trials by pressing the specified button.

Each trial involved one test and one reference car image. The reference car was superimposed on a background image, while the test car was placed on a grey background for comparison as shown in figure 2B. The background image for the reference stimulus was either intact or scrambled. The reference stimulus had a fixed size and position in the image. Its position corresponded to one of the road image’s two perceived distance positions (far or near). The test stimulus, on the other hand, had a fixed location at the centre of the grey background, and its size changed in logarithmic steps via key presses. The step size was halved after the first three response reversals to save scanner time.

Participants were asked to adjust the test car’s size to match the reference car’s size on the background image and then to press another button to register the size of the test car that looked the same as the reference. A subsequent trial was presented 0.1 s after the response was registered. There were 32 adjustment trials in total: (2 background images: intact and scrambled) × (2 rotations: upright and inverted) × (2 car positions: near and far) × (2 background image locations: left and right) × (2 starting sizes for the comparison car: smaller and larger than the reference). The conditions were presented in a random order.

#### Experimental runs

In the fMRI measurements of the illusion, we used similar stimulus conditions as the adjustment experiment, except for two differences. First, the background images and the car stimuli were placed at the horizontal centre of the screen. Second, we presented the car image only at the far position (correspondingly, the upper position in the upright-scrambled background condition) because pilot experiments showed a significantly larger perceptual effect when the car was at the far position, compared with the near position.

There were four stimulus conditions in a run: upright-intact, upright-scrambled, inverted-intact and inverted-scrambled. Since the far position corresponds to different screen locations for upright and inverted conditions, participants had to change their fixation between the two rotation conditions. To minimize the number of eye movements, we grouped intact and scrambled background conditions of the same rotation condition in the stimulus sequence.

**Figure 3.**
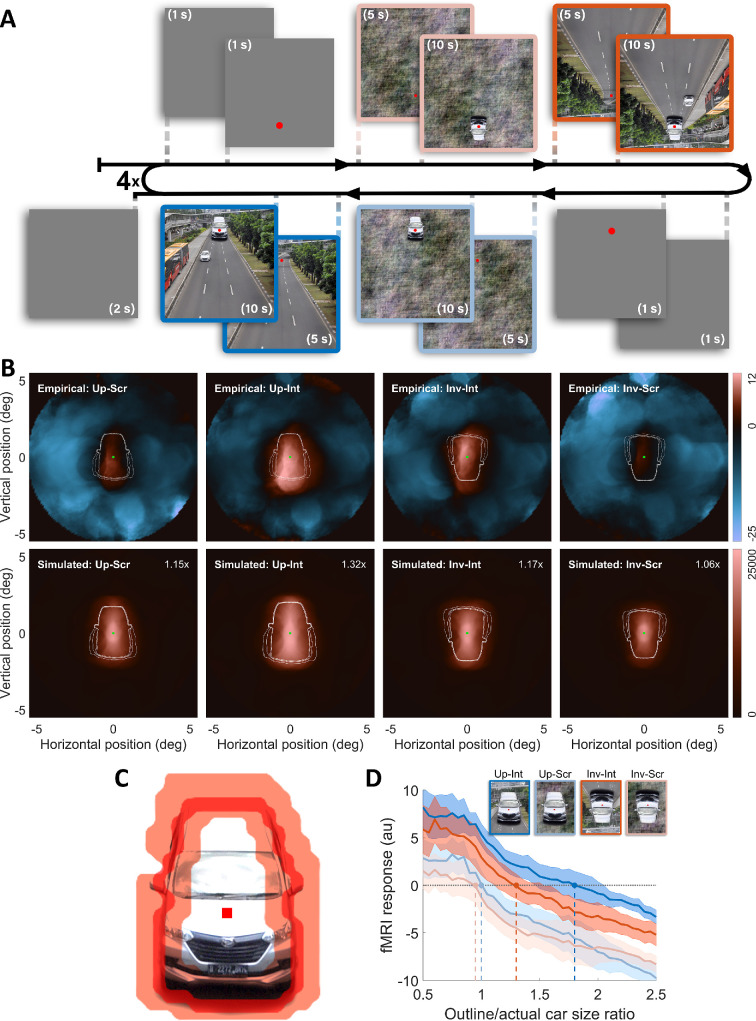
(A) Timeline of the fMRI experiment. (B). Back-projected V1 responses in the visual field. The upper row shows the back projections reconstructed from the observed data. White outlines show the to-scale outer edges of the presented car stimulus at different alpha channel thresholds (0.75, 0.5, 0.3, 0, respectively, from inner to outer). The green square represents the fixation point; warm and cold hues, respectively, represent positive and negative responses relative to the baseline. The bottom row displays the simulated back projections to varying car stimulus sizes, each panel showing the one that resulted in the best correlation with the observed projections of that condition. The relative sizes of the cars yielding this maximum correlation were written in the upper right corner of each plot, and outlines represent the sizes used in that projection. (C). The visual field ROI used in the analysis is shown with the red transparent area, which had a width of 0.5° around the edge of the car shape. The edge was illustrated with a red line. (D). Mean fMRI response as a function of car-shaped outline size relative to the actual car size averaged across participants. As the values in the *x-*axis increase, it represents the activation for more eccentric regions, corresponding to a larger car size. The shaded areas represent the standard error across participants. Vertical dashed lines indicate the zero-crossing points (the size ratio values where the average fMRI responses reach zero). Up: upright, Inv: inverted, Scr: scrambled, Int: intact.

The stimulus sequence (figure 3A) started with a one-second grey display, followed by a red fixation circle flickering on and off for 1 s, which served as a cue for the upcoming fixation location (i.e. the car location). The radius of the cue circle was 0.14${,}^{\circ }$ of the visual angle, twice as large as that of the regular fixation point. Following the cue, a background image of the corresponding condition was presented for 15 s, the last 10 s of which included the car image superimposed on the far position of the background. The car image flickered on and off at 3 Hz to ensure a strong fMRI response. The fixation point coincided with the geometric centre of the car image, and it was always present whenever there was a background image. Then, the following condition in the same fixation group appeared similarly (without the preceding fixation cue). The remaining two conditions of the other fixation position followed after the blank and cue displays. This sequence was repeated four times in a run, with a counterbalanced order of conditions. Lastly, a blank display was presented for 2 s at the end of the run. The order of conditions was predetermined and was different for all six runs.

Participants were required to remain fixated on the fixation point and to press a button whenever the fixation point changed colour from red to green. Colour changes lasted for 400 ms and occurred with a probability of 0.01 for every 400 ms period of each stimulus block.

#### Population receptive field mapping

We used bar stimuli in retinotopic mapping runs to estimate the pRFs [31]. A bar traversed a circular region of the visual field eight times, each time in a different direction. The initial sweep started from the bottom and moved upwards, with each subsequent sweep rotated $45^{\circ }$ clockwise from the previous one. Each sweep was completed in 25 s, consisting of twenty-four jumps by $0.59^{\circ }$ each. The bar width was $0.83^{\circ }$, with the height varying depending on its location. The bars reached their maximum height when they appeared in the centre of the circular region and were the shortest at the start and end of the sweeps. Bars contained a high-contrast checkerboard pattern flickering at 3.75 Hz. After the fourth and eighth sweeps, there were baseline periods of 25 s without any bar stimuli, including a fixation point and a dark-grey radar grid on the mid-grey background, which were also present in the rest of the run. Participants were instructed to maintain fixation on the fixation point and report colour changes. There was a 0.1 probability of colour change at every 200 ms. The colour change lasted for 200 ms.

#### (iii) MRI data acquisition

MR images were collected on a Siemens 3 Tesla scanner (Magnetom Trio, Siemens AG, Erlangen, Germany) with a 32-channel head coil.

T1-weighted structural images were obtained using the magnetization-prepared rapid acquisition with gradient echo (MP-RAGE) sequence with a TR of 2.6 s, a TE of 2.92 ms, a flip angle of $12^{\circ }$ and a 1 mm isotropic voxel size. High-resolution anatomical images were acquired to project the functional data onto. T2*-weighted functional images (268 images for the experimental runs and 260 images for pRF runs) were acquired with a 2 mm isotropic voxel size. Scans had a field of view of 192 mm, TR of 1 s, TE of 30 ms, flip angle of $62^{\circ }$, an rBW of 1680 Hz/pixel and a multi-band slice acceleration factor of 3. We used 36 slices angled to be approximately parallel to the calcarine sulcus. MR images for each participant were acquired in a single session.

#### (iv) Data analysis

#### Behavioural measurement of illusion

We recorded the size of the car stimuli that were perceived as equal to the reference stimulus. Here, the size was defined as the height of the smallest rectangle in pixels that contains the car image on the screen. We then calculated the ratio between the perceived and the actual stimulus sizes, averaged the ratios from the repeat trials of the same conditions and, finally, log-transformed the averaged ratios. We performed statistical analyses with the log-transformed ratios.

#### fMRI preprocessing

The functional images were preprocessed using SPM12 (Wellcome Centre for Human NeuroImaging; v. 7771). Motion artefacts were corrected using the realign and unwarp module with default parameters. The motion-corrected functional images were then co-registered with the structural image. Using FreeSurfer’s (v. 7.1.1) cortical reconstruction process, we converted the three-dimensional volumes into the inflated surface of the white and grey matter boundary by finding for each vertex the voxel at the middle position between pial and white-matter surfaces [32,33].

#### Population receptive field estimation

The mapping data were projected onto the surface mesh using FreeSurfer. The rest of the analysis was performed using SamSrf Toolbox (v. 9.032). Three runs of retinotopic mapping data were averaged after each vertex’s fMRI time series were linearly detrended and the z score normalized. Then, we calculated the noise ceiling for each vertex. The noise ceiling is an estimate of the reliability of visual responsiveness. It was calculated as follows: first, the time series of odd and even runs were correlated at each vertex. Then, using the Spearman–Brown prophecy formula [34,35], the reliability of the average of all three runs was calculated. Lastly, this measure was squared to obtain the maximum goodness of fit that can possibly be achieved for each vertex. More details of this calculation can be found in [36] and [37].

We performed the pRF analysis on the posterior portion of the cortex including the occipital lobe. We modelled the pRF as a two-dimensional Gaussian function. The Cartesian coordinates $x_{0}$ and $y_{0}$ defined the pRF location, and the standard deviation σ defined the pRF size. To estimate these parameters for each vertex, we first generated a binary representation of the stimulus movie for a given coordinate of the visual field, indicating whether or not a stimulus was present at a certain time (i.e. TR) and a coordinate. Then, we calculated each voxel’s predicted response based on a certain pRF profile and its overlap with the stimulus. The predicted responses were then convolved with the canonical haemodynamic response function (HRF) [38] to predict the blood oxygenation level dependent (BOLD) time series that would be observed with the three parameters of that pRF profile.

To find the optimal pRF parameters that minimize the error between the predicted and empirical BOLD time series, we employed a coarse-to-fine fitting strategy. First, we generated an extensive search grid with numerous plausible combinations of the pRF parameters. For each grid point, the predicted BOLD time series were correlated with the observed BOLD time series. The parameters yielding the highest correlation were then taken to the next stage, where they were further refined with the Nelder–Mead simplex search algorithm [39,40]. The optimum parameters were estimated based on the minimum squared residuals between the observed and the predicted BOLD time series. The response amplitude and the baseline parameters of the predicted time series were estimated via linear regression. We also calculated the goodness of fit, $R^{2}$, for each vertex, by comparing the predicted and observed time series and normalizing it by dividing $R^{2}$ by the noise ceiling.

The estimated pRF parameters were projected onto the cortical surface as polar angle and eccentricity maps. Then, these two maps were utilized to identify the regions of interest (ROI) for each hemisphere. We used the automated delineation tool of the SamSrf toolbox (v. 9.032). The tool drew a rough estimation of the visual area borders using the default atlas (Infernoserpent) within the toolbox. The auto-drafted delineations were manually adjusted and refined based on the literature [41–43].

Artifactual vertices were removed by denoising the pRF data. Denoising included removing the vertices, where (i) both $x_{0}$ and $y_{0}$ are zero, (ii) the sigma values were equal to or less than zero, and (iii) beta (amplitude) is smaller than 0.01 or greater than 3.

#### MRI measurement of illusion

For each participant, we specified a general linear model (GLM) in SPM12 (v. 7771). Events in each run were included in the model, together with each run’s six motion parameters as regressors. Other parameters were left default. Following the model estimation, we calculated four contrasts by subtracting the background-only blocks from the relevant car blocks to isolate the activation for cars at each rotation and background condition. Then we used Samsrf Toolbox (version 9.032) to project the GLM contrasts onto the cortical surface mesh for further analyses.

#### Back projection and MRI response simulation

We followed the steps outlined by Stoll *et al*. [30] to back-project the responses for each contrast onto the visual field. We first filtered V1 vertices and removed vertices in the GLM contrasts corresponding to the denoised pRF estimations in the same individual. Then, we pooled the responses from all participants based on the estimated $x_{0}$ and $y_{0}$ coordinates in the visual space. Then, we sampled the vertices responding to a given circular region (i.e. searchlight; radius = 1${,}^{\circ }$) in the visual field. The distance between each searchlight was 0.1${,}^{\circ }$. We averaged the responses falling within each searchlight and visualized the responses in the visual field space, as shown in figure 3B, upper row.

To evaluate the observed back projections better, we compared them to a set of back projections of simulated responses for varying sizes of the same stimulus. To achieve the simulated responses, we first generated a hypothetical set of car stimuli of varying sizes. We had 52 frames of different stimuli, each containing a scaled version of the car stimulus. We used the alpha channel of the actual car image, as the transparency values indicate where the stimulus was present. Stimulus sizes were logarithmically spaced, ranging from 0.5 to 4 relative to the actual size of the stimulus, and included finer steps between 0.8 and 1.5 for precision. For all of these stimulus sizes, we then predicted the response of V1 neurons based on the pRF estimates of each participant. Lastly, we pooled the predicted responses of all participants for each stimulus size and visualized them in the visual field using the searchlight procedure mentioned above. This process resulted in 52 simulated back projections for each hypothetical stimulus size. The bottom row of figure 3B shows one of these simulated back projections per condition.

#### Sliding window method

To analyse the extent of V1 activation for each condition, we used the sliding window method to sample responses within the edge-shaped region of 41 different car sizes that were linearly spaced between 0.5 and 2.5 times the actual size. The edge regions of all car images were defined as described in the electronic supplementary material. We then averaged the car size-binned responses of participants.

### (b) Results

#### (i) Perceptual effect

The results of the behavioural measurement are illustrated in figure 2B, separately for far and near positions. The y-axis represents the per cent size ratio, where perceptual overestimation and underestimation of the car size were shown with values larger and smaller than 100, respectively. For statistical analyses, however, we used the log-transformed ratio between the test and reference stimulus sizes to linearize the inherently nonlinear values. First, we tested if the perceived size of the car stimuli on the background image differs from its actual size. We found that the car stimuli seen on the intact background (both rotation conditions) were significantly perceived as larger than their actual sizes (upright, 175%: t(9)=6.5, p<0.001, inverted, 146%: t(9)=3.7, p=0.02, one sample t-tests, FDR correction applied). None of the other conditions survived multiple comparison correction (FDR, [44]), except for the far position of the upright-scrambled condition (t(9)=2.9, p=0.05).

We also tested the effect of car position (far and near), rotation (upright and inverted) and background (intact and scrambled) on the perceived size values. Results yielded a significant main effect of car position (F_1,9_ = 31.9, p<0.001), rotation (F_1,9_ = 11.5, p=0.008) and background (F_1,9_ = 38.5, p<0.001). In addition, all interactions were also significant (position × rotation, F_1,9_ = 6.5, p=0.03; position × background, F_1,9_ = 20.6, p=0.001; rotation × background, F_1,9_ = 11.8, p=0.008; position × rotation × background, F_1,9_ = 14, p=0.005).

#### (ii) Empirical and simulated back projections

First, we visually compared the empirical back projections for upright and the inverted intact (i.e. illusory) conditions with their corresponding scrambled (i.e. control) conditions. The first row of figure 3B represents the pooled V1 responses reconstructed in visual space for all four conditions. Since these back projections were obtained using the GLM contrasts, they indicate the activation for the car stimulus, isolated from its background. The warm (or cold) hues represent the regions in the visual field that correspond to an increase (or decrease) in the response compared with their respective baselines (response to the background only). Increased response associated with the car stimuli in the intact conditions spanned a larger region in the visual field (larger area of warm hues) compared with those in the control conditions. This was consistent with our expectations and also with the previous studies showing that V1 activity reflects the perceived size [16–18]. Importantly, we also observed a clear distinction between the back projections of the upright and inverted illusory (i.e. intact) conditions, reflecting the inversion effect we observed perceptually.

To quantify the difference between the observed back projections, we correlated them with the simulated back projections. Simulations were based on the predicted V1 response for a set of various scaled car stimuli. For each condition, we performed 52 two-dimensional correlations between the empirical back projection and the simulated back projections. The results showed that, for the upright-intact condition, the simulated back projection that resulted in the maximum correlation coefficient was obtained with a car stimulus that was 32% larger than the actual stimulus. For the inverted-intact condition, the maximum coefficient was observed with the car stimulus that was 17% larger than the stimulus presented in the experiment. The stimulus sizes that resulted in the best correlation in the scrambled conditions were much smaller than those for intact conditions, with respectively 15% and 6% larger stimuli for upright and inverted conditions (see electronic supplementary materials for visual comparison between the actual and simulated sizes yielding best correlation).

#### (iii) The extent of activation

To further investigate the effect of rotation and background on the fMRI response in terms of the spatial extent of activation, we computed the car-shape-binned responses using the sliding window method. The group average of the binned fMRI responses was plotted against the ratio between the car outline size used in each bin and the actual car size figure 3D. We defined the extent of activation as the zero-crossing point of the curves for each condition. The reason for using zero-crossing points was that zero on the y-axis represents no change in the activation for the main stimulus conditions (car + background) relative to the activation for their baseline condition (background only). Therefore, if the car stimulus in an illusory condition activates a larger cortical area compared with that in the control condition (scrambled), the zero-crossing points (of the car-size binned response curve) for the illusory condition should be shifted toward the right side on the x-axis, compared with the curve for the control condition. As shown in figure 3D, the curves and the zero-crossing points for the illusory conditions were positioned to the right of their corresponding control conditions. The extent of activation obtained by the sliding-window method reached approximately 1.3 and 1.8 on the x-axis, respectively, for the inverted-intact and upright-intact conditions. These should be interpreted in contrast to the zero-crossing points for the scrambled background conditions, which roughly equalled the actual car size, where the outline/actual car size ratio corresponded to one. Notably, the shift in the upright condition was larger compared with that in the inverted condition, congruent with the perceptual effect.

For statistical comparison, we bootstrapped individual curves 1000 times and recorded the zero crossing points of the average curve of each bootstrapped sample. We calculated the inversion effect by subtracting the zero-crossing points for the inverted condition from those for the upright condition. Similarly, we calculated the illusion effect by subtracting the scrambled condition from the intact condition, and the interaction effect by contrasting the illusion effect for both rotation conditions. The computed p-values showed a significant rotation effect for the intact background (p=0.004) but not for the scrambled background (p=0.06). The illusion effect was significant for upright conditions (p<0.001) and inverted conditions (p=0.004). There was no significant interaction between the background and the inversion effects (p=0.3).

#### (iv) Fixation performance

Participants, on average, responded to 93% of the fixation colour tasks within 1 s. Neither rotation (upright versus inverted, F_1,9_ = 0.002, p=0.97) nor background (intact versus scrambled, F_1,9_ = 0.82, p=0.39) showed significant main effects on the per cent fixation performance.

## 4. Discussion

We investigated the role of high-level processing in Ponzo-like size illusions by utilizing a simple yet potent manipulation: inversion of the pictures. Inversion weakened the illusion, with an exception discussed below, and critically, this inversion effect was reflected in V1 activation. We also observed stronger overall effects for realistic images. These findings show the involvement of high-level processing in perceiving object size in Ponzo-like illusions.

Reduced effect in the inverted images provides critical information about the visual processing of these illusions. While inverted images contain the same local, low-level features, such as relative size cues and angular perspective content, as their upright versions, they do not conform to the typical human experience of the visual environment. Therefore, a change in illusion magnitude should reflect the contribution of top-down processes, potentially a manifestation of an individual’s understanding of the fundamental size and distance relationship as presumably consolidated over constant exposure in real life. It is worth noting that although the inverted images do not match the typical visual diet, it is implausible that perceived depth would be cancelled out by inversion, especially in the case of realistic images. An inverted road should still be perceived as a road that recedes into the distance. However, the simplest backgrounds might become too ambiguous for the visual system when they are inverted, considering the limited depth cues and lack of prior experience with perspective cues converging at the bottom.

Remarkably, the inversion effect was also reflected in V1 activation. Our fMRI results for the upright condition replicated previous work by [17] and subsequent studies [16,18,19], which showed that neuronal activity in V1 reflects perceived rather than retinal size in Ponzo-like illusions. The perceptually larger stimulus activated a larger area in V1 compared with control conditions, which is also consistent with previous work on the moon illusion [45], afterimage size [46] and size adaptation [47]. Crucially, comparing the upright and inverted versions revealed that the spatial extent of activation was larger in the upright version. Since we posit that the inversion effect is based on top-down processing, our findings suggest that the neural correlate of the illusion in V1 must involve high-level processing that is being fed back from higher stages of the visual system.

Interestingly, inversion did not always reduce the illusion’s magnitude. The sky and the ceiling images, where the primary angular perspective cues were naturally upside-down, so to speak, replicated the illusion: the ostensibly farther object appeared larger than the nearer object, but inversion did not weaken the illusion. In fact, at least numerically, the inversion produced a stronger average illusion. Since the sky and ceiling images did not show an inversion effect similar to the road image, the inversion effect cannot be explained solely by typical expectations of scene orientation. Instead, these results, together with the strong correlation between the opposite rotation conditions of the road and ceiling images, as well as the road and sky images, suggest that the inversion effect might reflect a positional asymmetry in size constancy priors, potentially established over evolutionary time scales. Most depth and size judgements occur based on the cues on the ground, i.e. below the horizon. Ceilings, and especially the sky in the real world, convey less reliable sources of depth, leading size constancy to depend more on ground-based cues. This also aligns with other directional biases in visual perception, such as the light from above bias [48], and a recently reported up-bias [49]. A depth-inducing but otherwise ambiguous spiky pattern is more likely to be perceived as pointing upward rather than downward. Similarly, perceived depth in a round wavy geometrical shape reverses when the shape is inverted [50]. These biases might occur due to a prior for seeing near and far objects, respectively, at the bottom and top of the visual field.

Another directional bias in visual processing is the face inversion effect. Face recognition is typically more difficult when presented upside-down, compared to upright [51]. Duchaine *et al*. [52] demonstrated that extensive experience with upright faces contributes to this effect, along with an evolved mechanism for upright face preference. Their study of Claudio, a man with a congenital condition causing his head to be permanently positioned upside-down, showed no face inversion effect. This highlights the influence of visual diet on perceptual biases, indicating that prolonged exposure to certain visual orientations can shape perceptual mechanisms. Similarly, our findings on the asymmetrical effects of inversion in depth cues and size constancy could potentially reflect the influence of visual diet. However, our current data do not allow definitive conclusions about whether the modulation of this Ponzo-like illusion is due to experience or evolutionarily determined priors.

The decrease in the perceptual effect in the inverted Ponzo-like illusions has been reported by several studies [14,15], comparable to our findings. Our use of a wider range of stimuli, including a variety of visual environments and levels of depth cues, offered a more detailed examination of how inversion modulates the perceptual effect. We found that the magnitude of the upright illusion was weaker in the sky and ceiling images than in the road image. These versions may have differed from the road image, and also from one another, in terms of the available depth cues. The implied depth in the sky image was more ambiguous than in the ceiling image because of the possible alternative interpretations of the sky scene. One interpretation of the sky image was that the top plane was perceived as the nearer object (as in the ceiling condition). However, both planes can also be perceived to be at the same distance but at different altitudes. Such an additional ambiguity in the depth cues could explain the weaker illusion effect for the sky compared to the ceiling image. Similarly, we found weaker perceptual effects for simpler, abstract backgrounds than for photographic scenes. The abstract backgrounds contained limited depth information and lacked some features that would imply depth, such as textures, colour variation, shadows and lighting, compared with the realistic background condition. These findings suggest that the magnitude of the illusion decreases as the contextual information becomes more abstract, ambiguous and deviates from real-world experience, which is in line with earlier studies [11,13]. Accordingly, this elucidates the substantially stronger perceptual and neuronal effects we observed in our road condition, compared with those reported by previous studies that lacked photorealistic images (e.g. [3,17]).

One might argue that visual field differences introduced by inversion could confound our results. However, this concern is unfounded. In our fMRI experiment, we matched the retinotopic positions of the upright and the inverted car stimuli with the fixation point alternating between the top and bottom parts of the image. The shape of the car stimulus, however, was not symmetrical along the horizontal axis. Therefore the inversion introduced small differences between the upper and lower visual fields, which might be raised as a potential confound in our results. We considered whether the wider V1 activation in the upright condition, compared to the inverted, could be due to the stimulus covering a wider area in the lower visual field. Perceptual performance is better in the lower than the upper visual field (vertical meridian asymmetry; [53,54]). However, because our participants fixated the centre of the car stimuli, they only reached approximately 1.2° eccentricity. Previously reported visual field asymmetries occur at much more eccentric positions and increase with eccentricity [55]. Within the very centre of gaze, such asymmetries are probably negligible. Our back projection results also provided evidence for this: the back projections reflected the unique shape of the car stimulus proportionately in both inverted and upright conditions.

Could attention have any influence on our results? Fang *et al.* [19] showed that V1 activation in a Ponzo-like illusion was modulated by attention, such that there was no neural correlate of the illusion in V1 when the observers’ attention was directed away from the object. Although the attentional resources deployed to the scene might have been restricted by the presence of a fixation task, this could have only had a minimal effect in our study. The fixation task did not require a high attentional load, and there was no spatial separation between the fixation point and the car. Additionally, considering the small size of the car stimuli, we believe the attention was efficiently divided between the fixation task and the stimulus. However, this raises another question: Could the attentional engagement with the stimulus differ between the two inversion conditions? Such an assumption would predict fewer attentional resources were deployed for the inverted condition in our study, based on the results of [19]. We consider this highly implausible. A supposed attentional benefit for the upright conditions, which could lead to a larger V1 activation and a stronger perceptual effect might seem consistent with the results from the road condition, but this fails to explain the behavioural results for the ceiling and sky conditions which showed a reversed pattern of the inversion effect. Also, if anything, one would expect the inverted images to attract more attention due to the unfamiliarity of the scene. Given this inconsistency, the driving effect cannot be the attentional factors. Furthermore, behavioural performance on the fixation task, an index of attentional engagement, did not differ between conditions.

In conclusion, our study revealed that Ponzo-like size illusions engage high-level feedback mechanisms that incorporate contextual depth cues and visual experience, consequently modulating the neural representation of object size in V1.

## Data Availability

The behavioural data, fMRI data (where ethically permissible), stimulus-generation code and statistical analyses are publicly available at OSF [29]. Supplementary material is available online [56].
