# Correction to: ‘Perceiving object size in pictures involves high-level processing’ (2025), by Altan *et al.*

**DOI:** 10.1098/rspb.2025.2661

**Published:** 2025-11-26

**Authors:** Ecem Altan, H. Boyaci, Steven C. Dakin, D. Samuel Schwarzkopf

**Affiliations:** ^1^ School of Optometry and Vision Science, University of Auckland, Auckland, New Zealand; ^2^ Departments of Psychology and Neuroscience, Bilkent University, Ankara, Türkiye; ^3^ A.S. Brain Research Center & National Magnetic Resonance Research Center (UMRAM), Bilkent University, Ankara, Türkiye; ^4^ Department of Psychology, Justus Liebig University Giessen, Giessen, Hessen, Germany; ^5^ Institute of Ophthalmology, University College London, London, UK; ^6^ Experimental Psychology, University College London, London, UK

*Proc. R. Soc. B* **292**, 20242967 (Published online 14 May 2025). (https://doi.org/10.1098/rspb.2024.2967).

The description under ‘Population receptive field estimation’ (page 7 of PDF version) of how we calculated the noise ceiling of the fMRI data is inaccurate. Specifically, the sentence starting with ‘Lastly, this measure was …’ should instead read:

‘This measure is equal to the noise ceiling, the maximum goodness of fit that can possibly be achieved for each vertex.*’*

Note that this is only an error in the text that does not affect the results. We want to correct this statement to avoid incorrect usage of noise ceiling calculations by others. We unreservedly apologize for this incorrect statement.

